# Iron supplementation and deworming during pregnancy reduces the risk of anemia and stunting in infants less than 2 years of age: a study from Sub-Saharan Africa

**DOI:** 10.1186/s12884-023-05399-7

**Published:** 2023-01-25

**Authors:** Stanislav Seydou Traore, Yacong Bo, Guangning Kou, Quanjun Lyu

**Affiliations:** 1 Department of Nutrition and Food Hygiene, College of Public Health, Zhengzhou University, Zhengzhou, 450001 China; 2 Jockey Club School of Public Health and Primary Care, The Chinese University of Hong Kong, Hong Kong, 999077 China; 3 Centre of Sport Nutrition and Health, School of Physical Education, Zhengzhou University, Zhengzhou, 450001 China; 4 Department of Nutrition, the First Affiliated Hospital of Zhengzhou University, Zhengzhou, 450052 China

**Keywords:** Iron, Deworming, Stunting, Childhood anemia, Low birth weight, Infants, Sub-Saharan Africa

## Abstract

**Background:**

In sub-Saharan Africa, infant anemia, stunting and low birth weight remains major public health problems. It is unclear whether prenatal iron supplementation and/or deworming can reduce the risk of infant stunting, anemia and low birth weight. The aim of this study was to investigate the relationship between iron supplementation and/or deworming and stunting, anemia, and low birth weight in infants under two years of age in sub-Saharan Africa.

**Methods:**

Our studies examined pooled data from Demographic and Health Surveys (DHS) in twenty-three African countries collected between 2014 and 2020. childhood anemia and stunting in infants under the age of two were the primary outcomes. Iron supplementation and deworming during prenatal visits were the main exposure variables. A multivariate logistic regression model was used to investigate these relationships.

**Results:**

The prevalence of stunting was 29.9%, severe stunting was 10.6%, childhood anemia was 74.3%, childhood severe anemia was 3.2%, and low birth weight was 16.4%, respectively. The use of prenatal iron supplementation alone was associated with a significant reduction of childhood anemia [aOR (95% CI) = 0.9 (0.8–1.0)]. Prenatal deworming alone was associated with a significantly reduced risk of stunting [aOR (95% CI) = 0.7 (0.8–1.0)], childhood anemia [aOR (95% CI) = 0.7 (0.8–0.9)], and low birth weight [aOR (95% CI) = 0.7 (0.8–1.0)]. Prenatal iron supplementation plus deworming or iron supplementation (with or without deworming) were not associated with childhood anemia, infant stunting and low birth weight.

**Conclusions:**

In Sub-Saharan Africa, prenatal deworming alone has the potential to improve infants’ outcomes. Childhood anemia was improved with prenatal iron supplementation alone. Our recent findings indicate the necessity for prospective studies on the association between prenatal iron supplementation plus deworming and childhood anemia, stunting and low birth weight.

**Supplementary Information:**

The online version contains supplementary material available at 10.1186/s12884-023-05399-7.

## Background

The nutritional health of the woman, especially before and during pregnancy, is essential for the proper development of the infant [1]. The mother's diet must provide the appropriate nutrients for the harmonious development of the fetus and the infant throughout the first year of life [2]. Iron deficiency accounts for nearly half of all maternal anemia cases worldwide [3, 4], with the highest proportion occurring in Africa [5]. The main causes of iron deficiency are inadequate intake (e.g., low iron diet), increased iron requirements (e.g., during pregnancy), and chronic blood loss (e.g., severe hookworm infection) [6]. In 2019, approximately 45.8% of pregnant women in sub-Saharan Africa were anemic [7]. And more than 2 million people in the world are currently suffering from human helminth infections [8]. As part of prenatal care, the World Health Organization (WHO) had set standards of daily intake for iron at 30–60 mg and folic acid at 400 g to decrease the risk of maternal and neonatal anemia and adverse birth outcomes. In addition, a single dose of Albendazole (400 mg) or Mebendazole (500 mg) after the first trimester of pregnancy was strongly recommended for women living in areas with a high risk of worm infection [9, 10]. Since then, daily iron supplementation and deworming treatment (DWM) have become an integral part of antenatal care (ANC) practices in sub-Saharan African countries [11]. Anemia in women during pregnancy has been reported to increase the risk of infant stunting, childhood anemia and low birth weight [12, 13]. In Africa, stunting [14], childhood anemia [15–18], and low birth weight [19, 20] remain a public health problem to this day. The prevalence of stunting in infants under five years of age was 41% in 2020, anemia in infants was 60.2% in 2019, and low birth weight was 13.7% in 2019 [7, 21]. Iron supplementation has been shown to reduce the incidence of stunting [3, 4, 22], low birth weight [23] and childhood anemia [11, 24]. But some studies have not found such a relationship between prenatal iron supplementation and low birth weight [25] or stunting in infants [26]. However, it is unclear whether prenatal iron supplementation and/or deworming medications can reduce the risk of stunting, low birth weight, and anemia in infants younger than 2 years of age. Therefore, we used nationally representative surveys to investigate the association between prenatal iron supplementation and/or deworming medications and stunting, anemia, and low birth weight in infants under two years of age in sub-Saharan Africa.

## Materials and methods

### Data sources and study design

Our studies used nationally representative survey data from DHS in twenty-three Sub-Saharan African countries collected between 2014 and 2020. We used information from women's most recent live birth in the two years preceding the interview in each of these surveys. The DHS data were obtained from the DHS program website (http://dhsprogram.com/). Each survey gathered information on sociodemographic, health, and nutrition indicators from a nationally representative sample of households chosen through multistage cluster probability sampling. The survey procedures were thoroughly described [27]. In brief, the DHS survey data included prenatal iron supplementation, deworming medication use, antenatal care services use, presumed birth size, anthropometric data, and anemia status for the most recent live birth in the previous five years. We looked at a total of 36,879 mother-infant pairs (36,703 weighted) most recent live births 2 years prior to the interview, where anthropometric measurements were also available (Supplementary Table 1). The study included infants aged 6 months to 23 months and women aged 15 to 49 years. Any infants with missing anthropometric measurement data and any women with missing medication intake data (iron supplementation and deworming) were excluded from the analysis.

### Outcomes

Our main outcomes are listed and defined follows: stunting in infants: proportion of infants who have a z-score for height-for-age that is less than two standard deviations below the median of the 2006 WHO reference population [27]. Severe stunted infants had a z-score less than three standard deviations below the median [27]. Stunting is defined as a growth and development disorder that infants experience as a result of repeated infections and poor nutrition [27]. Childhood anemia: the proportion of infants aged 6 to 23 months with a hemoglobin level below 11 g per deciliter (g/dl). Childhood severe anemia is defined as a hemoglobin level below 7.0 g per deciliter (g/dl). Low birth weight: Birth weight reported as less than 2.5 kg based on a written record, coded "no" for weight greater than or equal to 2.5 kg and "yes" for weight less than 2.5 kg [27].

### Exposure variables and potential confounding factors

The ‘Iron supplementation (with or without deworming)’ variable was classified as yes or no depending on whether women took iron tablets or syrup during their most recent pregnancy. To investigate the impact of the number of days of iron intake on the outcomes, a variable was constructed. ‘Number of days of iron intake’ was defined as all women who had given birth in the previous two years and had taken iron tablets or syrup for at least two days during their past pregnancy, regardless of whether they received deworming medication. There were four categories for the variable: none, less than 60, 60 to 90, and more than 90 days of intake. The variable ‘Deworming (with or without iron supplementation)’ was classified as yes or no depending on whether women received deworming medication during their most recent pregnancy. Deworming treatment (Albendazole or Mebendazole) targets soil-transmitted helminthiases, of which the most common species infecting humans are Ascaris lumbricoides (roundworm), Trichuris trichiura (whipworm) and Ancylostoma duodenale or Necator americanus (hookworms) [9]. In addition, the variable "Iron supplementation and/or deworming" was added to explore the combined effect of iron and deworming on the study outcomes. It was defined as any women who took iron supplements alone (without deworming), deworming alone (without iron supplementation), or both (indicating those who received both iron supplements plus deworming medication), and was divided into four categories: none, iron supplementation alone, deworming alone, and iron supplementation plus deworming.

The covariates and potential confounders considered were the mother’s age (15–19, 20–29, 30–39, 40–49), Currently in union (Not in union, In union), Type of place of residence (Urban, Rural), Highest educational level (No education, Primary, Secondary, Higher), Wealth index (Poorest, Poorer, Middle, Richer, Richest), Number of ANC visits (None, < 4, >  = 4, don't know/missing), Improved Water Source (unimproved surface water, improved water), Sex of infants (Male, Female) and infant's age in months.

### Statistical analysis

SPSS version 24 was used for statistical analysis. All P-values were two-tailed, and statistical significance was defined as P ≤ 0.05. Using clusters, strata, and weight statements, all analyses were corrected for complicated survey design effects. The sampling weights defined/derived from the original national surveys were used. Descriptive statistics for participants were presented as means (Standard Error (SE)) for continuous and frequencies (percent) for categorical outcomes. We utilized multivariable logistic regression, which was adjusted for country, residence area, maternal marital status, maternal education levels, drinking water sources, household wealth index, mother age, infant’s sex, and infant’s age. Potential confounders were selected based on knowledge from previous studies [23].

## Results

In Sub-Saharan Africa (Table 1), half of the women were between the ages of 20 and 29, 35.1% had no education, 68% lived in rural areas, and 57.6% had attended four or more standard prenatal visits. Among infants under two of age, stunting was found in 29.9%, anemia in 74.3%, and low birth weight in 16.4%.

**Table 1:** Characteristics of women and their infants under the age of two in twenty-three Sub-Saharan African countries, based on pooled data

	Variables	Unweighted	Weighted	
		N	N	%
Age	15–19	3328	3205	8.7
	20–29	18,707	18,641	50.8
	30–39	12,290	12,348	33.6
	40–49	2554	2509	6.8
Currently in union	Not in union	5041	4925	13.4
	In union	31,838	31,778	86.6
Type of place of residence	Urban	11,288	11,740	32.0
	Rural	25,591	24,963	68.0
Highest educational level	No education	13,289	12,867	35.1
	Primary	12,902	13,021	35.5
	Secondary	9581	9616	26.2
	Higher	1106	1198	3.3
Wealth index	Poorest	9406	8282	22.6
	Poorer	8131	7969	21.7
	Middle	7542	7517	20.5
	Richer	6345	6895	18.8
	Richest	5455	6041	16.5
Number of ANC visits	None	3220	3217	8.8
	< 4	12,021	11,878	32.4
	> = 4	21,171	21,144	57.6
	don't know/missing	467	464	1.3
Improved Water Source	unimproved/surface water	11,039	10,546	29.7
	improved water	24,730	24,973	70.3
Sex of infant	Male	18,667	18,534	50.5
	Female	18,212	18,169	49.5
Infant's age in months^a^		14	14	5.0
Stunted	Yes	10,976	10,973	29.9
Severe stunted	Yes	3852	3882	10.6
Childhood anemia	Yes	27,542	27,285	74.3
Childhood severe anemia	Yes	1217	1184	3.2
Low birth weight	Yes	6055	5960	16.4

*ANC* Antenatal care

^a^mean and Standard deviation. N: number of mothers and infants’ pairs. %: percentage

More than two thirds of women (79.7%) had taken iron supplements (with or without deworming) during their last pregnancy, with 36.4% reporting use for 90 days or more (Table 2). 47.7% were dewormed (with or without iron supplementation) during pregnancy, and 43% of women received both iron supplementation and deworming.

**Table 2:** Prevalence of study exposure of most recent live births two years prior to the interview, Sub-Saharan Africa, 2013–2020

Variables	Unweighted	Weighted	
	N	N	%
Iron supplementation (with or without deworming)			
No	7421	7426	20.3
Yes	29,339	29,170	79.7
Number of days of the iron supplementation (with or without deworming)			
None	7421	7426	21.5
< 60 days	11,309	11,203	32.5
60–89 days	3474	3307	9.6
90 + days	12,330	12,568	36.4
Deworming (with or without iron supplementation)			
No	18,686	18,546	52.3
Yes	16,908	16,887	47.7
Iron supplementation and/ or Deworming intake			
None	5651	5645	16.0
Iron supplementation alone	13,005	12,871	36.4
Deworming alone	1617	1629	4.6
Iron supplementation plus Deworming	15,252	15,225	43.0

*N* number of mothers and infants’ pairs. %: percentage

**Fig. 1:**
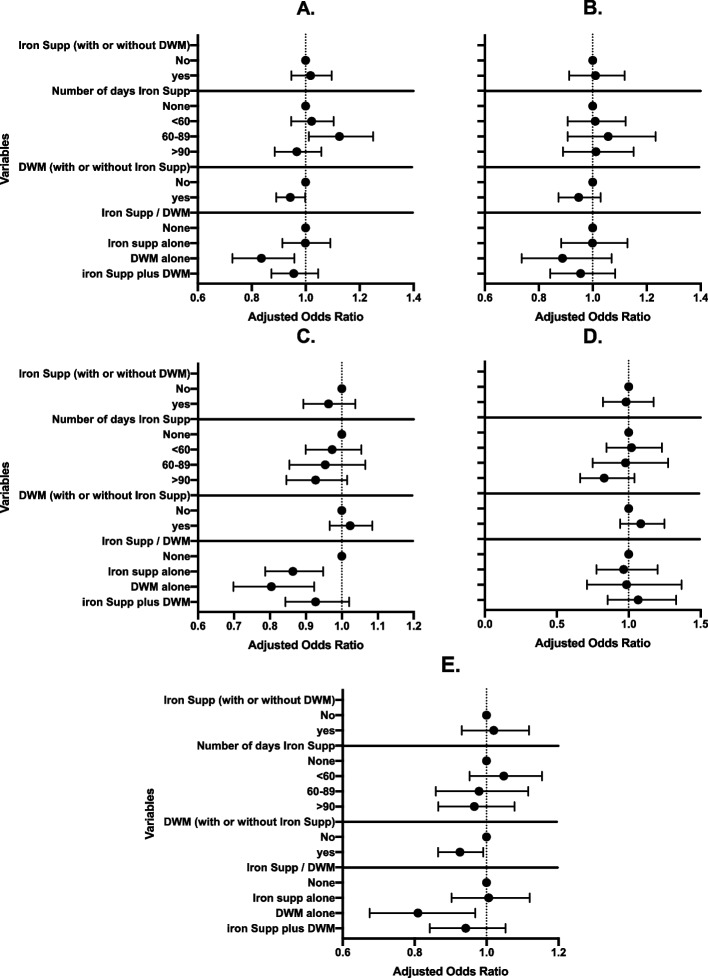
Effect of iron supplementation (with or without deworming), number of days of iron supplementation (with or without deworming), deworming (with or without iron supplementation), and combination of iron supplementation and/or deworming drug use on stunting, severe stunting, childhood anemia, childhood severe anemia, and low birth weight of infants under two years of age in sub-Saharan Africa. Iron Supp: Iron Supplementation, DWM: Deworming. **A** Stunting in infants, **B** Severe stunting, **C** Childhood anemia, **D** Childhood severe anemia, **E** Low birth weight

Figure 1 shows the findings of a multivariable logistic regression analysis of the prevalence of stunting in infants, severe stunting, childhood anemia, childhood severe anemia, and low birth weight. The risk of these five outcomes cited above did not reveal a significant reduction in infants whose mothers had used iron supplements (with or without deworming) during pregnancy when compared to infants whose mothers had not used iron supplements (with or without deworming) during pregnancy. On the other hand, Infants whose mothers had taken iron supplementation alone (had no DWM) had a significantly lower risk of childhood anemia [aOR (95% CI) = 0.9 (0.8–1.0)]. Infants whose mothers were only dewormed (had no iron supplementation) during pregnancy had a significantly lower risk of stunting, childhood anemia and low birth weight [aOR (95% CI) = 0.7 (0.8–1.0), aOR (95% CI) = 0.7 (0.8–0.9) and aOR (95% CI) = 0.7 (0.8–1.0), respectively] than those whose mothers were not dewormed.

## Discussion

This study, based on nationally representative data from 36,879 mother-infant pairs from 23 sub-Saharan African countries, found that 16.4% of children were born with low birth weight, 29.9% were stunted, 10.6% were severely stunted, 74.3% were anemic, and 3.2% were severely anemic, highlighting the persistence of these outcomes in this setting. Daily iron supplementation alone during pregnancy was associated with a reduced risk of childhood anemia. Similarly, deworming alone during pregnancy was associated with a reduced risk of infant stunting, anemia and low birth weight. In contrast, the use of iron supplementation plus deworming or iron supplementation (with or without deworming) for any duration was not associated with the above-listed five infant outcomes. To our knowledge, the current study is the first sufficiently powered study to report a protective effect of prenatal deworming on infant stunting in sub-Saharan Africa.

Our findings are consistent with those of China [28] and Nepal [29], in which iron and folic acid supplementation during pregnancy did not reduce the risk of infant stunting and low birth weight. In contrast, a recent analysis of pooled data from seven South Asian countries and Nepal [23, 30] showed that maternal use of iron and folic acid supplements during pregnancy significantly reduced infant stunting and low birth weight. Because folic acid has the advantage of accelerating the resolution of iron deficiency anemia in pregnant women [31], therefore the combination of iron and folic acid may have a stronger impact on infant outcomes in South Asia and Nepal. It is possible that the lack of effect of prenatal iron plus folic acid on the risk of infant stunting and low birth weight in China and Nepal [28, 29] is due to small sample size and insufficient statistical power. In addition, several studies that have shown a beneficial effect of iron and folic acid supplementation on the risk of low birth weight and/or stunting have also reported the importance of dose- and time-dependent effects, with supplementation in early pregnancy, over a long period of time and at a high dose being associated with a decreased risk [22, 32–34].

Our findings are consistent with previous studies in which deworming (with or without iron supplementation) during pregnancy reduces the risk of infant stunting [35, 36] and low birth weight [37]. One reason for our findings could be that reducing helminthic infections during pregnancy helps the woman absorb more nutrients [35]. Many previous studies have found a substantial association between the prevalence of intestinal parasites and an elevated risk of stunting [38–40] and low birth weight [37, 41]. In a developing country study [37], prenatal deworming was associated with a significant reduction in the risk of low birth weight in countries with a low prevalence of soil-transmitted helminths and a minor reduction in countries with a high prevalence. Prenatal deworming reduced the risk of low birth weight in infants weighing less than 1.5 kg in Peru [42] and Sri Lanka [43], but not in infants weighing less than 2.5 kg [44, 45].

Our findings are in contradiction with an Indian study in which iron supplementation (with or without deworming) during pregnancy reduces the risk of childhood anemia [46]. In India, the results may have been affected by the fact that older children (6 months to 5 years) were examined. In addition, our findings indicating that women who took prenatal iron supplementation alone or deworming alone had a reduction in childhood anemia are inconsistent with a recent study in sub-Saharan Africa [47] in which the risk of moderate/severe anemia was reduced in infants whose mothers had taken deworming alone, iron supplementation alone for ≥ 6 months, deworming + iron supplements for ≥ 6 months, and deworming + iron supplements for < 6 months, but not the risk of mild anemia. The latter study [47] had categorized anemia into mild and moderate/severe, which may have influenced the results. Women may be more likely to take one-time deworming drugs that have fewer negative effects than daily iron tablets, and only severely anemic women may be more likely to take iron supplements on a regular basis [48, 49]. The lack of anemia reduction in infants born to mothers who have taken iron supplements (with or without deworming) may be attributable to difficulties with antenatal care coverage and adherence, both of which are hampered by poor quality services, such as inadequate iron provision and inadequate counselling to encourage its appropriate use [50–54]. During pregnancy, the woman requires a large supply of iron to support the growth of the fetus and the placenta [46], and to allow the fetus to build up its own iron stores [1, 2]. Several studies have found a link between maternal hemoglobin, cord blood hemoglobin concentrations, and iron levels in breast milk [55, 56].

In contrast, our findings that prenatal deworming (with or without iron supplementation) does not have a beneficial effect on childhood anemia are consistent with a meta-analysis [57]. Because there are numerous causes of anemia [58], some of the benefits of parasite control may be negated if these other causes (such as inadequate supplementation, malaria, etc.) are not treated [59].

The study's strength lies in the use of pooled data from nationally representative surveys that offered a large sample of mother-infant pairs. The same standard methods were followed for the infant's anthropometric measures and hemoglobin testing. To reduce recall bias, live births from two years prior to the interview were chosen. There are several limitations to this study as well: we tried to reduce recall bias in our analyses by providing women samples of iron tablets/syrup to assist them to remember their prenatal iron intake. Other sorts of supplements, however, may be remembered by women. Because women were not randomly assigned to iron supplements, it's probable that confounding factors exist after our adjustments for several of the potential confounders.

## Conclusion

In Sub-Saharan Africa, prenatal deworming alone have the potential to improve infant’s outcomes. Childhood anemia was improved with prenatal iron supplementation alone. Strengthening health systems in countries with high prevalence of iron deficiency and parasitic infections; population health education to reduce behavioral barriers to accessing health services; and improving adherence and enabling initiation of iron supplementation as early as possible in pregnancy can all help to maximize the positive effects of these interventions. Our recent findings indicate the necessity for prospective studies in low- and middle-income countries to investigate effects of combined iron supplementation and deworming during pregnancy on childhood anemia, infant stunting and low birth weight.

## Supplementary Information

**Additional file 1: Supplementary Table 1.** Countries in Sub-Saharan Africa and the number of participants (mother and infant pairs) included in the study.

## Data Availability

Data Available at http://dhsprogram.com/.

## Abbreviations

DWM: Deworming
DHS: Demographic and Health Surveys
aOR: Adjusted Odds Ratio
CI: Confidence interval
WHO: World Health Organization
ANC: Antenatal care
