# Supplementary material for: Iron supplementation and deworming during pregnancy reduces the risk of anemia and stunting in infants less than 2 years of age: a study from Sub-Saharan Africa
Source: BMC Pregnancy Childbirth. 2023 Jan 25;23:63. doi: 10.1186/s12884-023-05399-7 (PMC9875517; doi:10.1186/s12884-023-05399-7)
Supplement: Supplementary file 1 — Additional file 1: Supplementary Table 1. Countries in Sub-Saharan Africa and the number of participants (mother and infant pairs) included in the study. [file 12884_2023_5399_MOESM1_ESM.docx]

Iron Supplementation And Deworming During Pregnancy Reduces the Risk of Anemia And Stunting in Infants Less Than 2 Years of Age: A Study from Sub-Saharan Africa

**Supplementary Table 1.** Countries in Sub-Saharan Africa and the number of participants (mother and infant pairs) included in the study

| Countries | Year of survey | Unweighted | weighted | |
| --- | --- | --- | --- | --- |
|  |  | N | N | % |
| Angola | 2015-16 | 1799 | 1683 | 4.6 |
| Benin | 2017 | 1896 | 1903 | 5.2 |
| Burundi | 2016-17 | 1842 | 1905 | 5.2 |
| Congo | 2013-14 | 2193 | 2120 | 5.8 |
| Cameroun | 2018 | 1273 | 1322 | 3.6 |
| Ethiopia | 2016 | 2490 | 2761 | 7.5 |
| Ghana | 2014 | 786 | 758 | 2.1 |
| Gambia | 2019 | 1106 | 1030 | 2.8 |
| Guinea | 2018 | 999 | 961 | 2.6 |
| Liberia | 2019-20 | 821 | 718 | 2 |
| Lesotho | 2014 | 440 | 435 | 1.2 |
| Mali | 2018 | 1222 | 1314 | 3.6 |
| Malawi | 2015-16 | 1596 | 1580 | 4.3 |
| Nigeria | 2018 | 3305 | 3358 | 9.1 |
| Rwanda | 2014-15 | 1091 | 1109 | 3 |
| Sierra Leone | 2019 | 1271 | 1257 | 3.4 |
| Senegal | 2017 | 3274 | 3038 | 8.3 |
| Togo | 2013-14 | 986 | 949 | 2.6 |
| Tanzania | 2015-16 | 2854 | 2849 | 7.8 |
| Uganda | 2016 | 1251 | 1241 | 3.4 |
| South Africa | 2016 | 264 | 275 | 0.7 |
| Zambia | 2018 | 2680 | 2637 | 7.2 |
| Zimbabwe | 2015 | 1440 | 1499 | 4.1 |
| Pooled Data | 2013-2020 | 36879 | 36703 | 100 |

N: number of mother and infant pairs. %: percentage.
